# Editorial: Ethics in Psychiatry and Psychotherapy

**DOI:** 10.3389/fpsyt.2021.742218

**Published:** 2021-08-11

**Authors:** Cynthia M. A. Geppert, Rebecca Weintraub Brendel, Manuel Trachsel

**Affiliations:** ^1^University of New Mexico School of Medicine, Albuquerque, NM, United States; ^2^Massachusetts General Hospital and Harvard Medical School, Boston, MA, United States; ^3^Center for Bioethics, Harvard Medical School, Boston, MA, United States; ^4^Institute of Biomedical Ethics and History of Medicine, University of Zurich, Zurich, Switzerland; ^5^Clinical Ethics Unit, University Hospital Basel and University Psychiatric Clinics, Basel, Switzerland

**Keywords:** psychiatry, psychotherapy, ethics, autonomy, coercion, digitalization, decision-making capacity, informed consent

The multifaceted and multidisciplinary field of ethics is relevant to any practitioner of psychiatry and psychotherapy. There is hardly another branch of medicine that has, from its very emergence as a specialty, raised such profound and complex ethical questions as the fields of psychiatry and psychotherapy (1, 2). Traditional ethical issues in psychiatry and psychotherapy include the value judgments inherent in the irreducibly subjective aspects of the processes of formulating a diagnosis and setting treatment goals. Other ethical questions in psychiatry and psychotherapy are related to involuntary commitment, coercion, or autonomy in patients whose psychiatric disorders may compromise decisional capacity and hence the ability to provide informed consent, the therapeutic relationship, privacy, confidentiality, therapeutic boundary violations, multiple relationships, and any form of exploitation. In recent years, new ethical questions have arisen related to dramatic changes in treatment modalities, exponential growth in neuroscience, and major shifts in social attitudes toward mental health and its most distinctive and essential values. These novel ethical challenges facing psychiatrists and psychotherapists range from the uses of new techniques, such as deep brain stimulation and the impact of evolving concepts of psychiatric genetics, to the role of online interventions, clinical palliative care for individuals with mental illness, or peer support in treatment. These are just a few examples of ethical issues in psychiatry and psychotherapy, and for the present Special Topic, we welcomed contributions spanning the landscape of this broad field to capture its depth and complexity and also included not only empirical but also conceptual papers. As a result, the Special Topic now captures the diversity of interest and expertise in psychiatric and psychotherapeutic ethics.

Two articles address neuroscience and the Cartesian mind-body problem, transmuting it into mind-brain dualism. Glannon examines the ethical implications for treatment of this current critical tension in psychiatry between seeing mental illnesses alternatively as disorders of the mind or of the brain and the implication of this practice for patients. He argues, instead, that neuroscience research has demonstrated the interdependency of mental and neural processes in maintaining mental health and causing mental illness and, therefore, that as an ethical matter this artificial dualistic thinking can cause harm to patients by limiting therapeutic interventions. The corollary is that dualistic thinking “can limit therapeutic interventions for patients suffering from major psychiatric disorders” and Glannon therefore concludes that “taking the full extent of mind-brain interaction into account is […] ethically imperative in psychiatric research and practice.” In response to Glannon's argument, Schleim critiques the persistence of mind-brain dualistic language in philosophical and scientific discourse for its perpetuation of a reductionism. Contrary to Glannon's assumption, Schleim contends that patients are quite willing to embrace neuroscientific explanations of psychiatric illness and may instead underestimate the value of psychotherapy. In rejecting dualist in favor of mechanistic and biopsychosocial explanations that take levels of description and understandings into account, Schleim suggests that we can achieve integrative formulations and approaches to advance the treatment of mental illness.

Approaching ethics from the vantagepoint of empirical study and machine learning, Yao et al. report a cross-sectional study in which they used machine learning and an online survey in We Chat to predict negative side effects from psychotherapy as a means of isolating factors that influence the emergence of unwanted events perceived during psychotherapy. In the 370 online questionnaire responses analyzed, negative emotions such as anxiety and anger were the most common side effects experienced in psychotherapy and the patient's perception of the therapists' own emotional state during the therapy was the most accurate predictor that the patient would experience these negative effects. The authors conclude that machine learning may assist therapists in identifying side effects of therapy that are often overlooked so that they may be addressed constructively.

While Yao et al. embrace the promise of neurotechnology, Stanghellini and Leoni in their exploration of digital phenotyping instead highlight the threat it may represent to integrity and authenticity. In this study, they collected and analyzed quantitative data from personal electronic devices such as mobile phones to identify clinical factors that could be utilized to clarify diagnosis and target treatment. The authors caution that this form of digital psychiatry may substantively and adversely alter bodily experience, violate the privacy of psychophysical space, and reformulate conceptions of humanity and the relationality that grounds it without adding explanatory power to psychiatric etiology.

More traditional ethics dilemmas such as the exercise of coercion are also represented in the present Special Topic. The paper by Efkemann et al. discusses the development and empirical validation of a German version of the Staff Attitude to Coercion Scale (SACS). While the original version included a 3-factor structure consisting of critical, pragmatic, and positive staff attitudes toward coercion, German translation required a change to an instrument with a one-factor structure constituting rejection or approval of coercion, which was achieved and validated. The authors emphasize the importance of this work to advance the use of validated instruments that measure attitudes toward coercion in order to reduce coercive clinical treatment interventions.

Münch et al. examine whether John Stuart Mill's maxim about the harm principle can form the basis of a diagnosis in the case of pedophilia and antisocial personality disorder. They contend that in DSM-5 and ICD-10, the criterion for both disorders is harm to others rather than the harm to self that is the standard for most diseases in psychiatry and medicine. The authors claim that these classifications rely more on moral judgments of what is socially unacceptable or labeled criminal than scientific criteria. They present arguments for and against keeping the current conceptualizations of the disorders in future classification systems and conclude with a recommendation that harm to others should not constitute a diagnosis unless there is also distress or dysfunction experienced by the acting individual.

The article from Bieber et al. explores the key ethical domains of parental autonomy, decision-making capacity, and consent as they arise in the care of children and adolescents with mental disorders. They report on two cases: one a youth with an eating disorder, and the other a young patient with schizoaffective disorder. In each case, the decisional capacity of the parents to understand the young person's diagnosis and based on that understanding to make appropriate treatment choices is questionable. The authors conclude that in cases where the risk of imminent harm may be low yet concern for medical neglect remains, a formal evaluation of parental capacity within the frame of a systematic review of ethical principles can help guide decision making in this challenging area and fulfill clinicians' beneficence-grounded obligations.

This reflection on consent and decision-making capacity reminds us that one of the most significant contributions of bioethics to medicine and psychiatry is the importance of patient autonomy. Three articles in this Special Topic take a closer look at its ethical importance for the psychotherapeutic alliance. Gerger et al. offer a theoretical and ethical analysis of the key characteristics that constitute “Good Psychotherapy” arguing that ethical values call for an expansion of the patient's role in psychotherapy. They conclude that therapists should facilitate this greater participation through a more personalized and activated informed consent process that empowers patient decision making.

Blease et al. explain how sharing “Open Notes” in psychotherapy is yet another means of promoting patient self-determination and enhancing informed consent in psychotherapy. Health care systems and professionals are increasingly utilizing “Open Notes” which are electronic records patients can access usually through specialized patient portals and often in near real-time. The authors contend that “Open Notes” will enhance relational autonomy, foster patient's procedural knowledge of psychotherapy and improve patient recall and engagement while still safeguarding professional autonomy.

Nestoriuc et al. report on their study to modify informed consent in order to reduce nocebo effects. They assessed the effect of providing information on the nocebo effect to patients on patients' desire for knowledge about antidepressant side effects. Of 97 patients recently prescribed antidepressants and randomized to the nocebo information or education about the history of antidepressants. Those patients who received the nocebo information wanted to know less about side effects and more about mechanisms and placebo effects than the history group. The authors suggest that these results could potentially improve treatment participation and reduce side effect experience and reporting.

Two articles highlight the diverse contexts and persons encountered in psychiatric ethics and the many types of psychotherapeutic interventions available. Amado et al. share their retrospective study of 2 to 9 year outcomes following tailored cognitive remediation (CR) provided as part of a personalized psychosocial rehabilitation program. Acknowledging the low employment rate of those with serious mental illness, they sought to identify effective interventions for this group with historically low employment rates. The study showed that CR was beneficial to employment and subjective well-being, with effects persisting as much as 9 years after therapy.

An international perspective is provided in an article from Kizilhan and Neumann who focus on the principle of justice in psychotherapy for patients who have suffered trauma from war or other humanitarian crises. Their central question was how psychotherapy can contribute to the restoration of justice in individuals who have suffered violence, displacement, and myriad injustices. The authors compellingly argue that “if war has a negative impact on health, then programs that focus on justice, peace, and stability should be able to offset or reduce this negative impact.” They set out ethical standards and principles to inform new approaches to psychotherapy with traumatized populations based on human rights, and thereby contribute to efforts for achieving social and political justice for survivors.

Two final articles in the collection outline practical approaches to translate ethical values and virtues into treatment to improve the health and lives of patients with mental illness. Gerritsen et al. discuss how the clinical ethics support service (CESS) approach of moral case deliberation (MCD) can aid forensic psychiatrists moving toward contact-based care where boundary and safety concerns are paramount. MCD is a structured conversation method where professionals with the help of a facilitator engage in critical reflection on difficult moral questions in the practice of forensic psychiatry.

Finally, Haltaufderheide et al. examined CESS, which has been relatively underutilized in psychiatry compared to medicine. The results of their 13 semi-structured interviews with members of CESS and the mental health professionals who consult them illustrate the types of problems and expertise involved in psychiatric CESS. They propose an empirical taxonomy of dyadic, triangular, and systemic ethics concerns noting that CESS focuses mostly on the first two types of problems. Further, professionals and CESS members have different understandings of the CESS expertise and responsibility especially for the third type. This suggests the need for CESS members to attune their solutions more closely to the problems for which practitioners request support, and to develop a stable professional identity.

The 14 articles in this Special Topic offer a fascinating tour of the variety of ethical issues encountered in psychiatry and psychotherapy that the editors hope will inspire readers to take further journeys into the field.

## Author Contributions

CG wrote the first draft of the manuscript, and MT and RB critically revised it. All authors read and approved the final version.

## Conflict of Interest

The authors declare that the research was conducted in the absence of any commercial or financial relationships that could be construed as a potential conflict of interest.

## Publisher's Note

All claims expressed in this article are solely those of the authors and do not necessarily represent those of their affiliated organizations, or those of the publisher, the editors and the reviewers. Any product that may be evaluated in this article, or claim that may be made by its manufacturer, is not guaranteed or endorsed by the publisher.
